# Human Mitochondrial DNA Polymerase Metal Dependent UV Lesion Bypassing Ability

**DOI:** 10.3389/fmolb.2022.808036

**Published:** 2022-03-09

**Authors:** Joon Park, Noe Baruch-Torres, Shigenori Iwai, Geoffrey K. Herrmann, Luis G. Brieba, Y. Whitney Yin

**Affiliations:** ^1^ Department of Biochemistry and Molecular Biology, University of Texas Medical Branch, Galveston, TX, United States; ^2^ Sealy Center for Structural Biology and Molecular Biophysics, University of Texas Medical Branch, Galveston, TX, United States; ^3^ Laboratorio Nacional de Genómica para la Biodiversidad, Centro de Investigación y de Estudios Avanzados del Instituto Politécnico Nacional, Irapuato, Mexico; ^4^ Division of Chemistry, Graduate School of Engineering Science, Osaka University, Toyonaka, Japan

**Keywords:** mitochondrial DNA, DNA polymerase gamma, UV lesion, metal-dependence, TLS

## Abstract

Human mitochondrial DNA contains more UV-induced lesions than the nuclear DNA due to lack of mechanism to remove bulky photoproducts. Human DNA polymerase gamma (Pol γ) is the sole DNA replicase in mitochondria, which contains a polymerase (*pol*) and an exonuclease (*exo*) active site. Previous studies showed that Pol γ only displays UV lesion bypassing when its exonuclease activity is obliterated. To investigate the reaction environment on Pol γ translesion activity, we tested Pol γ DNA activity in the presence of different metal ions. While Pol γ is unable to replicate through UV lesions on DNA templates in the presence of Mg^2+^, it exhibits robust translesion DNA synthesis (TLS) on cyclobutane pyrimidine dimer (CPD)-containing template when Mg^2+^ was mixed with or completely replaced by Mn^2+^. Under these conditions, the efficiency of Pol γ′s TLS opposite CPD is near to that on a non-damaged template and is 800-fold higher than that of exonuclease-deficient Pol γ. Interestingly, Pol γ exhibits higher exonuclease activity in the presence of Mn^2+^ than with Mg^2+^, suggesting Mn^2+^-stimulated Pol γ TLS is not via suppressing its exonuclease activity. We suggest that Mn^2+^ ion expands Pol γ′s *pol* active site relative to Mg^2+^ so that a UV lesion can be accommodated and blocks the communication between *pol* and *exo* active sites to execute translesion DNA synthesis.

## Introduction

Ultraviolet (UV) exposure of DNA causes dimerization of two adjacent pyrimidines, forming cyclobutane pyrimidine dimers (CPDs) or (6-4) pyrimidine-pyrimidones ((6-4)PP), which stalls replicating polymerases (Varghese and Wang, 1967a; Varghese and Wang, 1967b; Brash and Haseltine, 1982). Human mitochondria lack nucleotide excision repair or photolyase that removes the photoproducts (Maher et al., 1976; Maher et al., 1979; Maher et al., 1982); therefore, mitochondrial DNA replication machinery would inevitably encounter the UV photoproducts. Increasing sun exposure is correlated to a large spectrum of mitochondrial DNA (mtDNA) deletions in dermis, which, albeit unknown in mechanism, has been used as a biomarker for UV exposure and chronological aging (Birch-Machin et al., 1998; Kawasaki et al., 2000; Ray et al., 2000; Harbottle et al., 2010; Bharti et al., 2014). Deletions and mutations of mtDNA are also associated with skin cancers (Durham et al., 2003; Yang et al., 2004).

Human mitochondria DNA is replicated by DNA polymerase gamma (Pol γ), which consists of a catalytic subunit Pol γA and a dimeric accessory subunit Pol γB. Pol γ possesses activities of 5′-3′ polymerization (*pol*) for DNA synthesis, 3′-5′ exonuclease (*exo*) for proofreading, and 5′-deoxyribose phosphate lyase for DNA repair. Pol γB has no intrinsic enzymatic activity, but it accelerates polymerization rate, increases affinity to DNA, and enhances processivity of the holoenzyme (Johnson et al., 2000; Johnson and Johnson, 2001; Lee et al., 2010). Pol γA belongs to the A-family polymerases that are single polypeptide enzymes structurally resembling a right-hand shape and containing fingers, palm, and thumb domains (Steitz, 1999; Garcia-Diaz and Bebenek, 2007; Bębenek and Ziuzia-Graczyk, 2018). Other replicative A-family DNA polymerase members include bacterial DNA polymerase I and phage T7 DNA polymerase.

All known DNA polymerases require divalent metal ions to catalyze DNA synthesis. A general mechanism of two-metal-ion catalysis established a foundation for DNA and RNA polymerases’ enzymatic reactions (Steitz and Steitz, 1993). An exonuclease-deficient Pol γ variant was shown to have limited translesion DNA synthesis (TLS) activity on UV lesion-containing DNA in the presence of Mg^2+^, suggesting that Pol γ possesses intrinsic TLS ability (Kasiviswanathan et al., 2012). Mitochondria are the main storage site of intracellular Mn^2+^ ion (Maynard and Cotzias, 1955; Liccione and Maines, 1988; Gavin et al., 1990; Gavin et al., 1992), which raises an important question on impact of Mn^2+^ or Mg^2+^/Mn^2+^ mixture on Pol γ mtDNA replication as well as TLS.

We report here studies of the A-family member Pol γ lesion bypassing across UV lesions in the presence of different metal ions. We show that Pol γ is unable to replicate past the CPD- or (6-4)PP-containing DNA template in the presence of Mg^2+^, but displays efficient TLS ability comparable to that on the non-damaged template in the presence of Mn^2+^ or Mg^2+^/Mn^2+^ mixture. Mn^2+^ promotes TLS activity without involvement of the polymerase’s exonucleolytic activity. The Mn^2+^-mediated *trans*-UV lesion DNA synthesis appears uniquely to mitochondrial DNA polymerase and is absent in other A-family DNA polymerases tested. These results also give a novel insight into the metal-regulated error recognition and communication between the *pol* and *exo* active sites in Pol γ.

## Results

### Mn^2+^ Ion Stimulates Robust Translesion DNA Synthesis Across CPD but Not (6-4)PP

**FIGURE 1:**
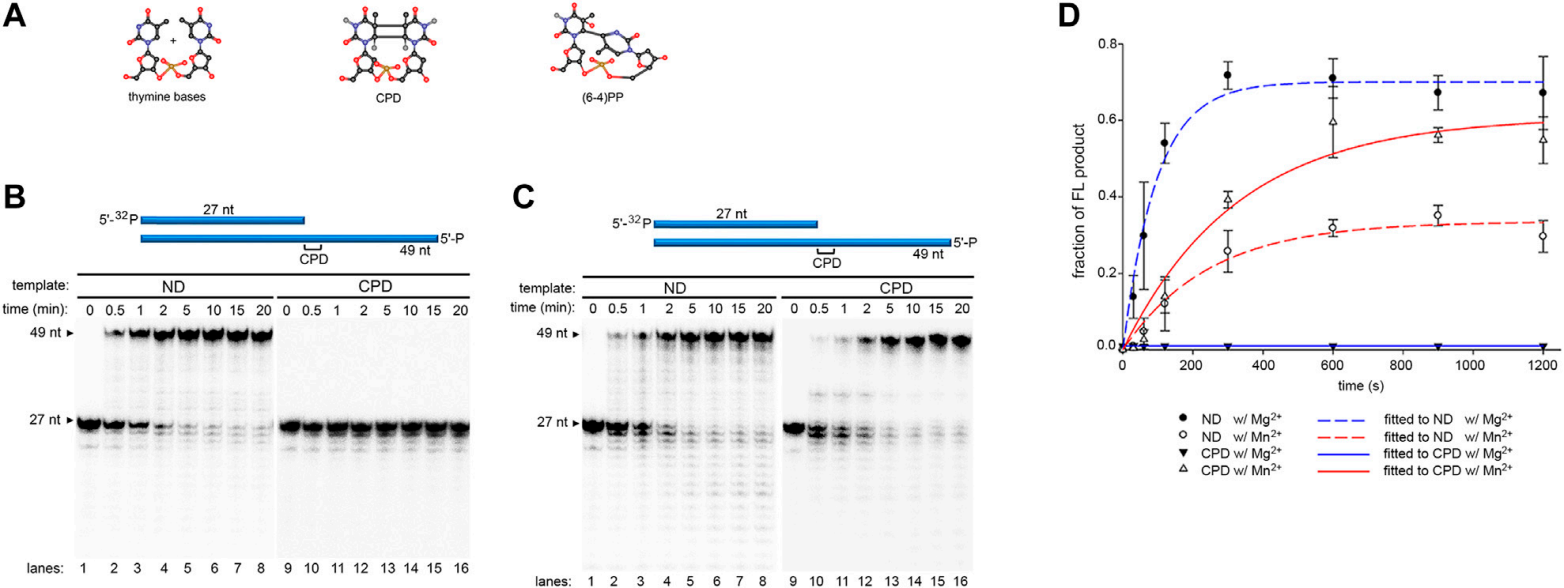
DNA synthesis of wild-type Pol γ on non-damaged and CPD-containing templates in the presence of Mg^2+^ or Mn^2+^. **(A)** Structure of thymine bases, CPD, and (6-4)PP where nitrogen is represented in blue spheres, oxygen in red spheres, carbon in black spheres, and phosphorus in golden spheres. **(B)** Time-dependent DNA synthesis of wild-type Pol γ on 5′-^32^P-Primer N (P_N_) annealed to a non-damaged template (T-ND) (*lanes* 1–8) or CPD-containing template (T-CPD) (lanes 9–16) in the presence of Mg^2+^. **(C)** Time-dependent DNA synthesis of wild-type Pol γ on 5′-^32^P-P_N_ annealed to T-ND (lanes 1–8) or T-CPD (lanes 9–16) in the presence of Mn^2+^. **(D)** Quantification of normalized full-length (FL) products from **(B)** and **(C)** over time in seconds. The results are presented as mean and standard deviation from three independent experiments.

DNA templates (49 nt) containing either a CPD (T-CPD) or (6-4)PP (T-(6-4)PP) (Table 1) at the 28th position from the 3′-end was synthesized as described in Methods. Pol γ translesion replication was first tested on a 27 nt primer (P_N_) annealed to the T-CPD where the 3′- thymine (3′- T) in the CPD serves as the coding base (Figure 1A, *lanes* 9–16, Table 1). Time-dependent (0–20 min) primer extension assay was conducted in the presence of Mg^2+^. As a control, a parallel assay was carried out on a non-damaged template (T-ND) with identical sequence except that the CPD is replaced by 2 dT (Figure 1A, *lanes* 1–8, Table 1). In anticipation of higher polymerase activity on T-ND, the Pol γ concentration was reduced (see Methods). For convenience, only the full-length product formation rate at normalized enzyme concentration was used for rate calculation. Full-length formation rate of Pol γ on T-ND was 4.5 nM/s (Figure 1C). However, on T-CPD, the primer was not extended, but instead was degraded to P_N-1_ to P_N-3_ over time (Figure 1A *lanes* 9–16).

**TABLE 1:** Sequences of DNA templates and primer.

Name	Sequence
P_N_	5′-AGC TAT GAC CAT GAT TAC GAA TTG CTT-3′
T-ND	3′- TCG ATA CTG GTA CTA ATG CTT AAC GAA TTA AGC ACG TCC GTA CCA TCG A-5′
T-CPD	3′- TCG ATA CTG GTA CTA ATG CTT AAC GAA **T<>T**_**CPD**_A AGC ACG TCC GTA CCA TCG A-5′
T-(6–4)PP	3′- TCG ATA CTG GTA CTA ATG CTT AAC GAA **T<>T**_**6-4**_A AGC ACG TCC GTA CCA TCG A-5′

To evaluate the effect of high Mn^2+^ content in mitochondria on Pol γ activity, we substitute Mg^2+^ with Mn^2+^ and repeated the above experiments. In stark contrast to results with Mg^2+^, Pol γ exhibited strong TLS across CPD in the presence of Mn^2+^ (Figure 1B, *lanes* 9–16). The rates of full-length product formation by Pol γ on T-CPD were calculated to be 0.58 nM/s, which is only 1.7-fold lower than that on T-ND at 1 nM/s (Figure 1C). Interestingly, Pol γ is 4.5-fold less efficient on the non-damaged template in the presence of Mn^2+^ than in the presence of Mg^2+^.

**FIGURE 2:**
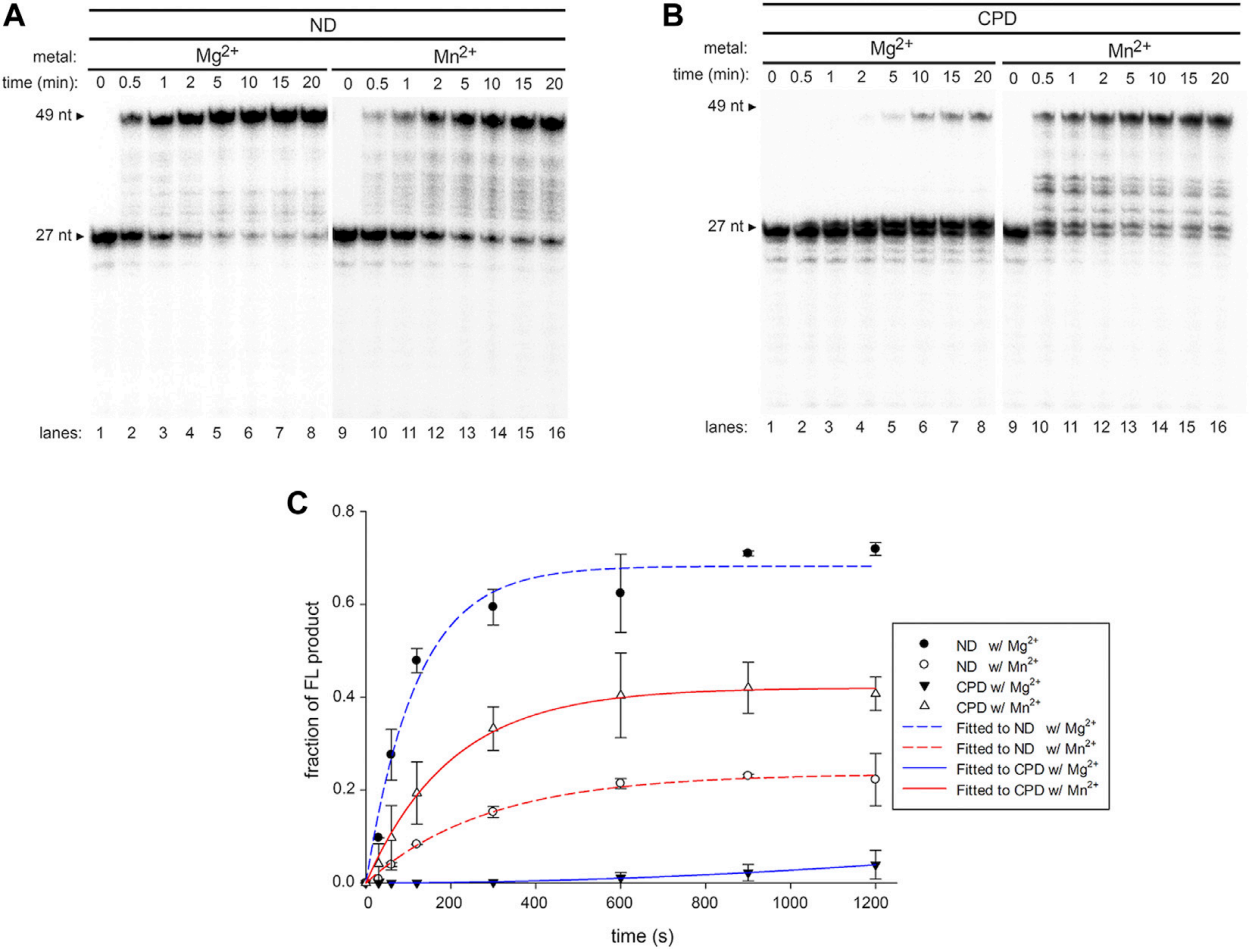
DNA synthesis of exonuclease-deficient Pol γ on non-damaged and CPD-containing templates in the presence of Mg^2+^ or Mn^2+^. **(A)** Time-dependent DNA synthesis of exonuclease-deficient (*exo*-) Pol γ on 5′-^32^P-P_N_ annealed to T-ND in the presence of Mg^2+^ (lanes 1–8) or Mn^2+^ (lanes 9–16) **(B)** Time-dependent DNA synthesis of Pol γ *exo*-on 5′-^32^P-P_N_ annealed to T-CPD in the presence of Mg^2+^ (lanes 1–8) or Mn^2+^ (lanes 9–16). **(C)** Quantification of normalized full-length (FL) products from **(A)** and **(B)** over time in seconds. The results are presented as mean and standard deviation from three independent experiments.

Previous studies showed that exonuclease-deficient (*exo*-) Pol γ displayed limited intrinsic CPD-bypassing ability (Kasiviswanathan et al., 2012). To assess whether Mn^2+^ plays a redundant role with exonuclease activity in Pol γ TLS activity, Pol γ *exo*- (D198A/E200A) (Spelbrink et al., 2000) was analyzed on T-ND and T-CPD. In the presence of Mg^2+^, Pol γ *exo*- extended a small amount of the primer P_N_ over the CPD site to full-length product, consistent with the previously reported data (Kasiviswanathan et al., 2012). However, a predominant amount of primer was extended by only a single nucleotide and formed product P_N+1_ (Figure 2A, *lanes* 1–8). Accumulation of the P_N+1_ indicates that silencing exonuclease enables the polymerase to synthesize across the 3′-T but not 5′-T of the CPD, suggesting that synthesis using the 5′-T as a coding base is the rate-limiting step of Pol γ TLS.

In the presence of Mn^2+^, Pol γ *exo-* exhibited significantly elevated TLS activity (Figure 2B, *lanes* 9–16). The rate of Pol γ *exo-* full-length product formation on T-CPD was calculated to be 0.81 nM/s with Mn^2+^, which is more than 800-fold increase than that with Mg^2+^ (0.001 nM/s) and exceeds that of wild-type (0.58 nM/s) (Figure 2C), confirming that suppressing *exo* activity indeed increases TLS in the presence of either metal ion. P_N+1_ product was drastically diminished, indicating Mn^2+^ facilitates Pol γ to overcome the energy barrier at the rate-limiting step. Similar to wild-type enzyme, Mn^2+^ exhibits inferior activity (eightfold lower) on Pol γ *exo*- synthesis on T-ND than Mg^2+^.

**FIGURE 3:**
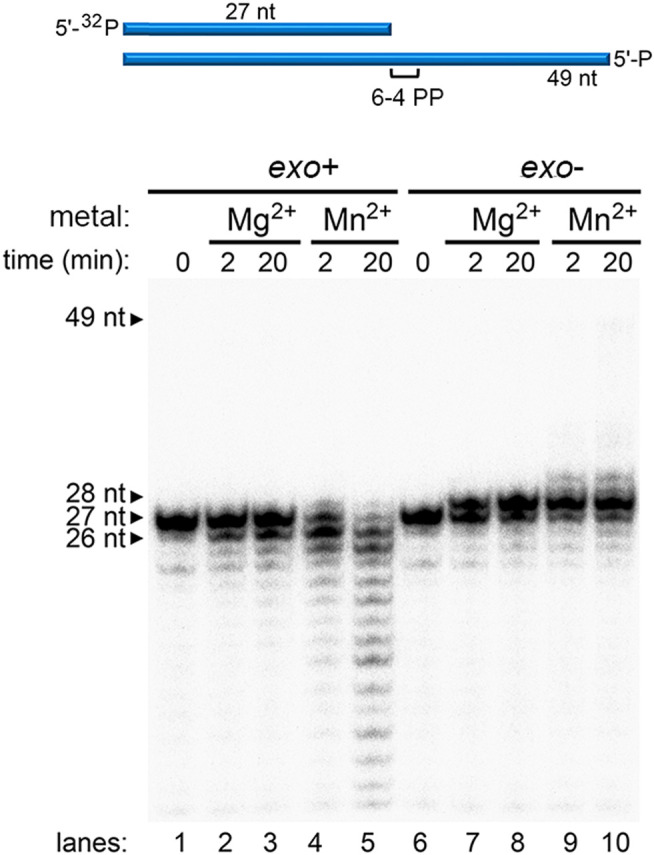
Metal-dependent DNA synthesis of wild-type and exonuclease-deficient Pol γ on a (6-4)PP-containing template. Time-dependent polymerization assay of wild-type Pol γ (*exo*+) on 5′-^32^P-P_N_ annealed to T-(6–4)PP in the presence of Mg^2+^ (lanes 2 and 3) or in the presence of Mn^2+^ (lanes 4 and 5) and of Pol γ (*exo*-) on identical DNA substrates in the presence of Mg^2+^ (lanes 7 and 8) or Mn^2+^ (lanes 9 and 10).

When translesion synthesis was tested on (6-4)PP lesion containing template, wild-type Pol γ showed no activity in the presence of either Mg^2+^ or Mn^2+^ (Figure 3, *lanes* 4 and 5). However, Pol γ *exo*- extended the primer to P_N+1_ with Mg^2+^, similarly to that on the T-CPD (Figure 3, *lanes* 7 and 8). Similarly, no full-length product was formed in the presence of Mn^2+^, and only P_N+1_ and a small amount of P_N+2_ was formed (Figure 3, *lanes* 9–10). Taken together, these results show that exonuclease activity contributes to the Pol γ′s lack of TLS activity, and Mn^2+^ further promotes Pol γ′s TLS ability independent of exonuclease activity.

### Mn^2+^ Stimulation of Pol γ TLS Is Independent of Exo Activity

**FIGURE 4:**
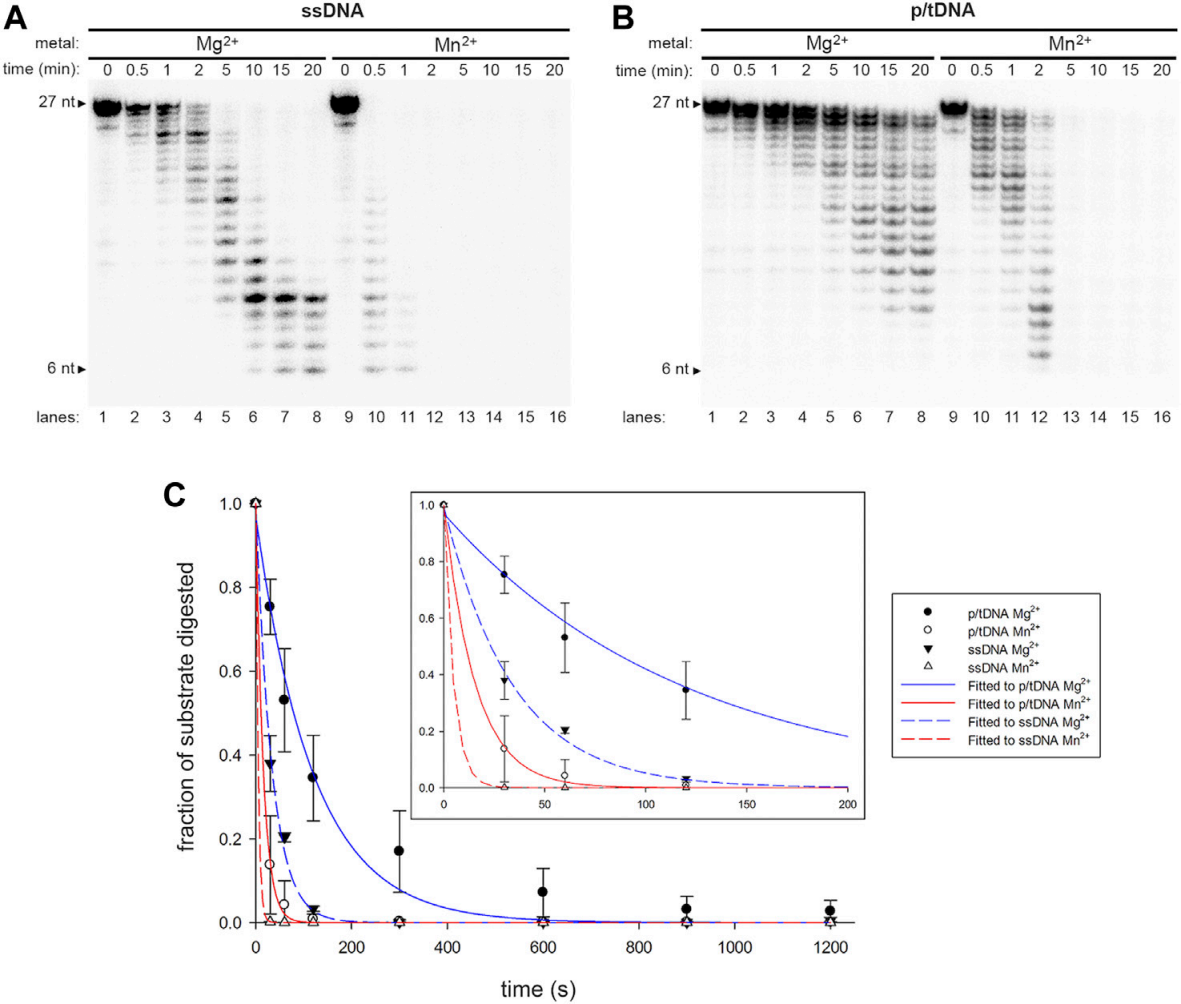
Metal-regulated exonuclease activity of wild-type Pol γ. **(A)** Exonuclease assay of Pol γ on a single-stranded DNA, 5′-^32^P-P_N_, in the presence of Mg^2+^ (lanes 1–8) or in the presence of Mn^2+^ (lanes 9–16). **(B)** Exonuclease assay Pol γ on primer/template, 5′-^32^P-P_N_/T-ND, in the presence of Mg^2+^ (lanes 1–8) or in the presence of Mn^2+^ (lanes 9–16) **(C)**. Quantification of normalized fraction of substrate remaining in **(A)** and **(B)** over time in seconds shown. The results are presented as mean and standard deviation from three independent experiments. Shorter time points (0–200 s) are shown as an inset.

The results thus far showed Mn^2+^ promotes TLS ability to both Pol γ wild-type and *exo-* variant, suggesting Mn^2+^ functions independent of exonuclease activity. To test the conclusion, we tested the *exo* activity of Pol γ with Mn^2+^ and compared it to that with Mg^2+^. We defined the rate of exonucleolysis as disappearance of the substrate and exonuclease events following the initial reaction were discounted, thus it is an underestimate of the actual rate. The rate of exonucleolysis of Pol γ on single-stranded DNA P_N_ is 12.6 min^−1^ with Mn^2+^, sevenfold faster than that of Mg^2+^ at 1.8 min^−1^ (Figures 4A,C). Pol γ *exo* activity was also assayed on duplex P_N_/T-ND (Figure 4B). The excision rate of the duplex P_N_/T-ND in the presence of Mn^2+^ is at 3.9 min^−1^, which is about eightfold higher than Mg^2+^ at 0.5 min^−1^ (Figure 4C). Our results are consistent with a previous report where Mn^2+^ stimulates porcine liver Pol γ excision of terminal mismatch (Longley and Mosbaugh, 1991). As Mn^2+^ increased *exo* activity of Pol γ on P_N_/N-ND, which is free of error, it suggests that primer is sent more frequently from the *pol* to the *exo* site. As suppressing *exo* activity as well as addition of Mn^2+^ that increases *exo* activity both promote Pol γ′s TLS, we predict that Mn^2+^ must reduce error recognition ability of reading correct Watson-Crick base pairing geometry, which in turn promotes erroneous shuttling of the primer strand to the *exo* site.

### Pol γ CPD Bypassing Activity in the Presence of Metal Ions Mixture

**FIGURE 5:**
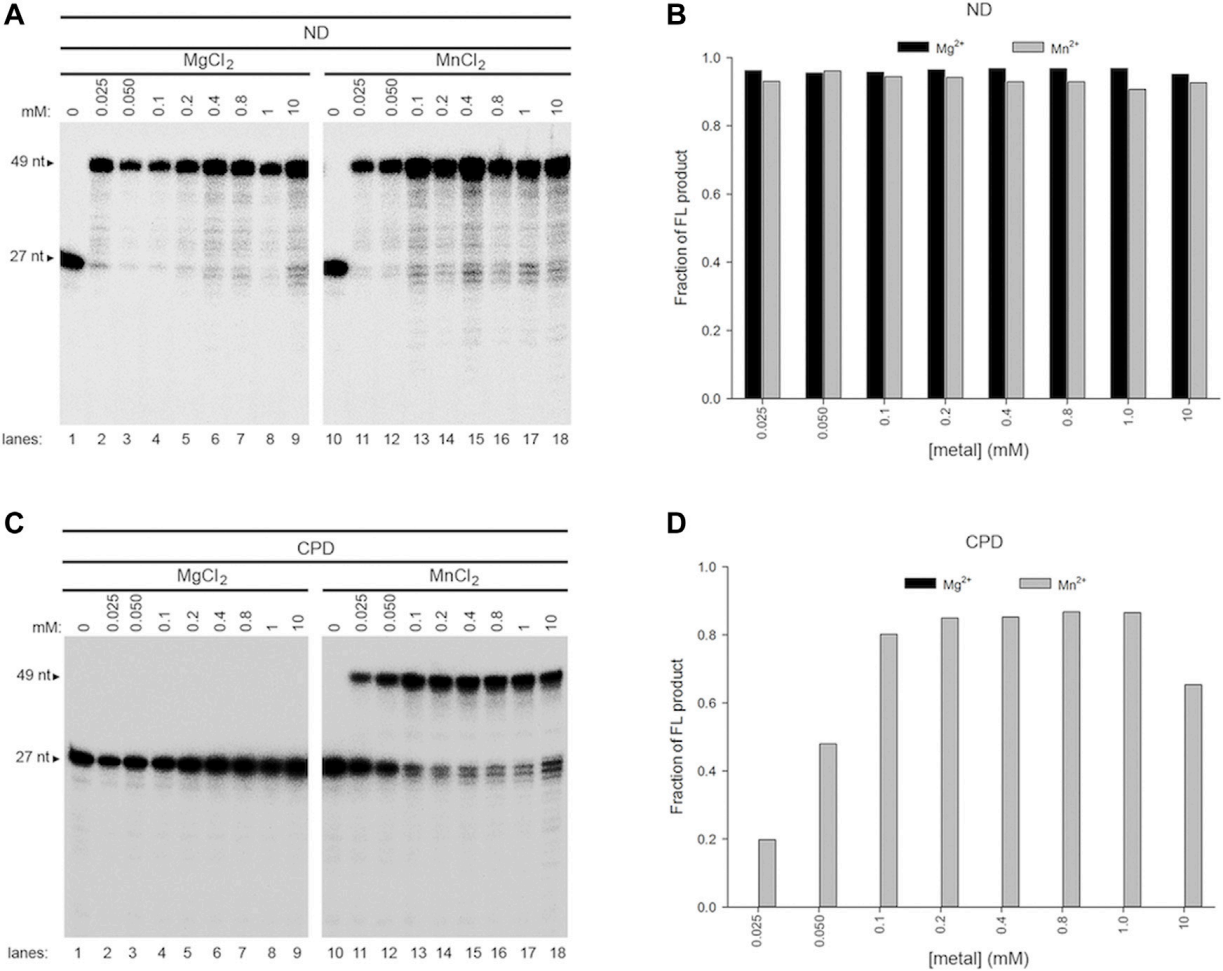
Effect of a single metal ion on wild-type Pol γ DNA activity on non-damaged and CPD-containing templates. **(A)** Wild-type Pol **γ** primer extension on T-ND annealed to 5′-^32^P-PN in the presence of Mg^2+^(lanes 1–9) or Mn^2+^ (lanes 10-18). **(B)** Quantification of the full-length product on T-ND vs. a gradient of Mg^2+^ or Mn^2+^ shown in **(A)**. **(C)** Wild-type Pol **γ** primer extension on T-CPD annealed to 5′-^32^P-PN in the presence of Mg^2+^ (lanes 1–9) or Mn^2+^ (lanes 10-18). **(D)** Quantification of the full-length product on T-CPD vs. a gradient of Mg^2+^ or Mn^2+^ shown in **(C)**.

**FIGURE 6:**
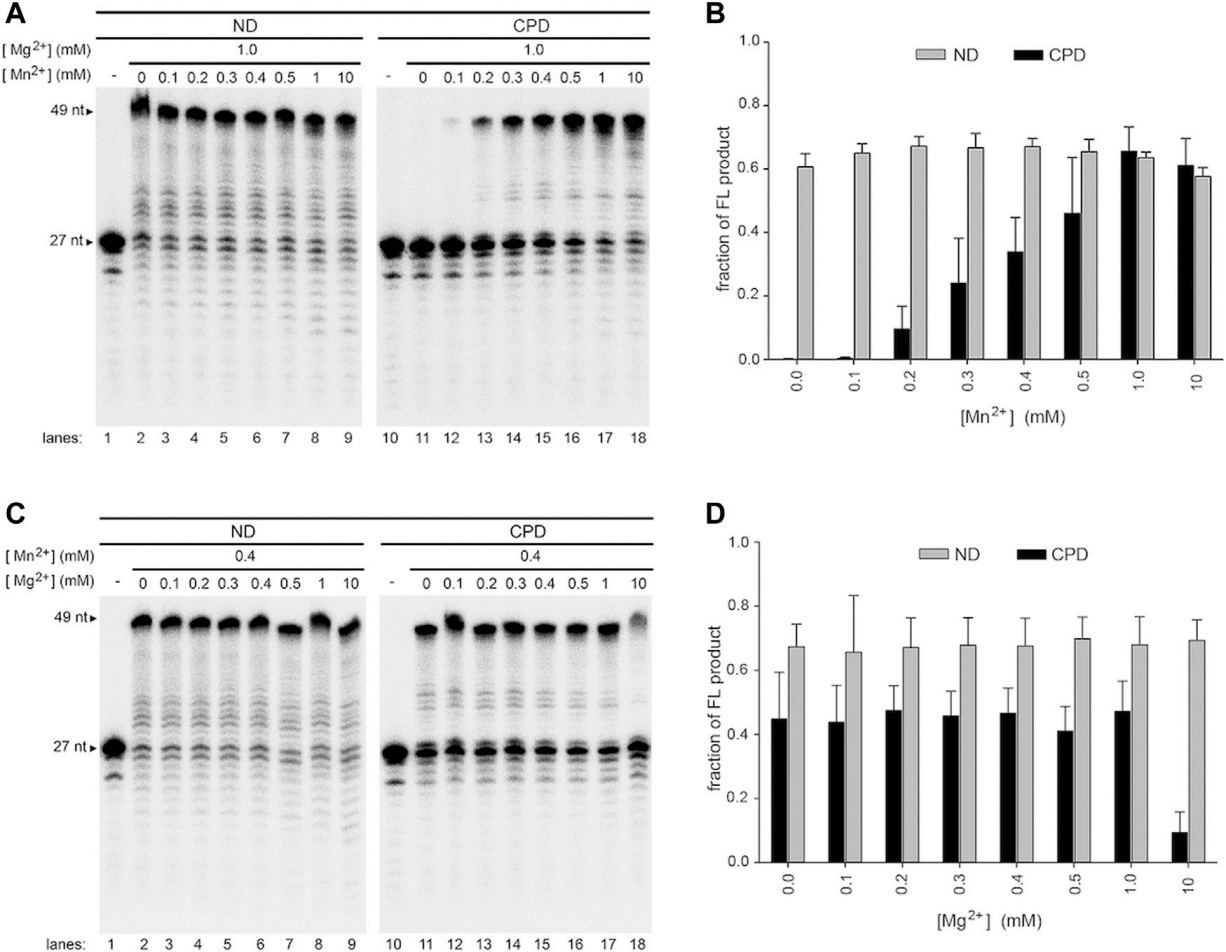
Effect of mixed metal ions on wild-type Pol γ DNA synthesis on non-damaged and CPD-containing templates in a mixture of Mg^2+^ and Mn^2+^. **(A)** Wild-type Pol γ primer extension on T-ND (lanes 1–9) or T-CPD (lanes 10–18) annealed to the 5′-^32^P-P_N_ at a constant concentration of Mg^2+^ and varied concentrations of Mn^2+^. **(B)** Quantification of the full-length product vs. concentration of Mn^2+^ shown in **(A)**. **(C)** Wild-type Pol γ primer extension on T-ND (lanes 1–9) or T-CPD (lanes 10–18) annealed to the 5′-^32^P-P_N_ at constant concentration of Mn^2+^ and varied concentrations of Mg^2+^. **(D)** Quantification of the full-length product vs. concentration of Mg^2+^ shown in **(C)**.

We show that Mg^2+^ and Mn^2+^ play opposite roles on Pol γ TLS. Since both ions are present in mitochondria, we tested the effects of metal ion mixture. Pol γ′s translesion synthesis assays were carried out at a fixed, near physiological concentration of one metal ion and varying the concentrations of the other. Specifically, Mg^2+^ was kept constant at 1.0 mM, and Mn^2+^ varied from 0.1 to 10 mM (Figure 6A); Mn^2+^ was kept constant at 0.4 mM, and Mg^2+^ varied from 0.1 to 10 mM (Figure 6C). Titrations of a single metal were also performed (Figure 5). As each dNTP is capable of binding to one of either metal ion and with higher affinity to Mn^2+^, the precise concentrations of free Mg^2+^ and Mn^2+^ ions for catalysis cannot be accurately calculated, but, in the presence of a total 0.8 mM dNTPs, it should be significantly lower than the initial concentrations.

In the presence of Mg^2+^ alone, Pol γ showed no TLS activity on CPD at any concentration tested (Figure 5C, lanes 1-9, **5D**), whereas, in the presence of Mn^2+^ alone, it produced full-length (FL) TLS product from 0.025 to 10 mM (Figure 5C, lanes 10–18, **5D**).

The TLS activity with mixed metal ions differs from that with a single metal ion alone. For example, assays with constant 1.0 mM Mg^2+^ and increasing Mn^2+^, Pol γ began to show TLS activity at 0.1 mM Mn^2+^ (Figure 6A, lane 10–18, **6B**). Nonetheless, different from Pol γ producing 97% FL product with 0.1 mM Mn^2+^ on T-ND (Figure 5B), it only produced 5% FL TLS product with 0.1 mM Mn^2+^/1.0 mM Mg^2+^ on T-CPD (Figure 6B); the polymerase produced 95% FL product with 0.2 mM Mn^2+^ alone on T-ND (Figure 5B) but no activity with 1.0 mM Mg^2+^ on T-CPD (Figure 5C, lanes1-9), yet producing 12% FL TLS product with metal mixture (Figure 6B). These results suggest that Pol γ′s metal binding sites are unlikely occupied by the same metal ion. We thus hypothesize that both metals are bound to the polymerase simultaneously.

A similar conclusion can be drawn from assays with fixed Mn^2+^ and varying Mg^2+^. In the presence of 0.4 mM Mn^2+^ and substoichiometric (0.1–0.3 mM), equimolar (0.4–0.5 mM) to slightly excess stoichiometric among Mg^2+^ (1.0 mM), Pol γ generated 35–40% FL TLS product (Figure 6C, lanes 10–17, **6D**). Even with the addition of 25-fold excess, 10 mM Mg^2+^, Pol γ still displays 10% of TLS activity (Figure 6C, lane 18, **6D**), suggesting high probability of Pol γ binding to both metal ions.

We should note that under the reaction condition Pol γ TLS activity was observed (0.2 mM Mn^2+^ and 1.0 mM Mg^2+^, Figure 6A, lane 13), and assume dNTPs exhibit equal affinity to Mg^2+^ and Mn^2+^ ions and ignore metal binding to DNA, the free Mn^2+^ ions available for Pol γ catalysis is ∼67 µM {0.2−0.8∗[0.2(1+0.2)]} (mM), below the reported physiological concentration of Mn^2+^ in mitochondria (Corkey et al., 1986; Romani, 2011).

### Mn^2+^ Does Not Stimulate Translesion Synthesis in Other Family A Polymerases

**FIGURE 7:**
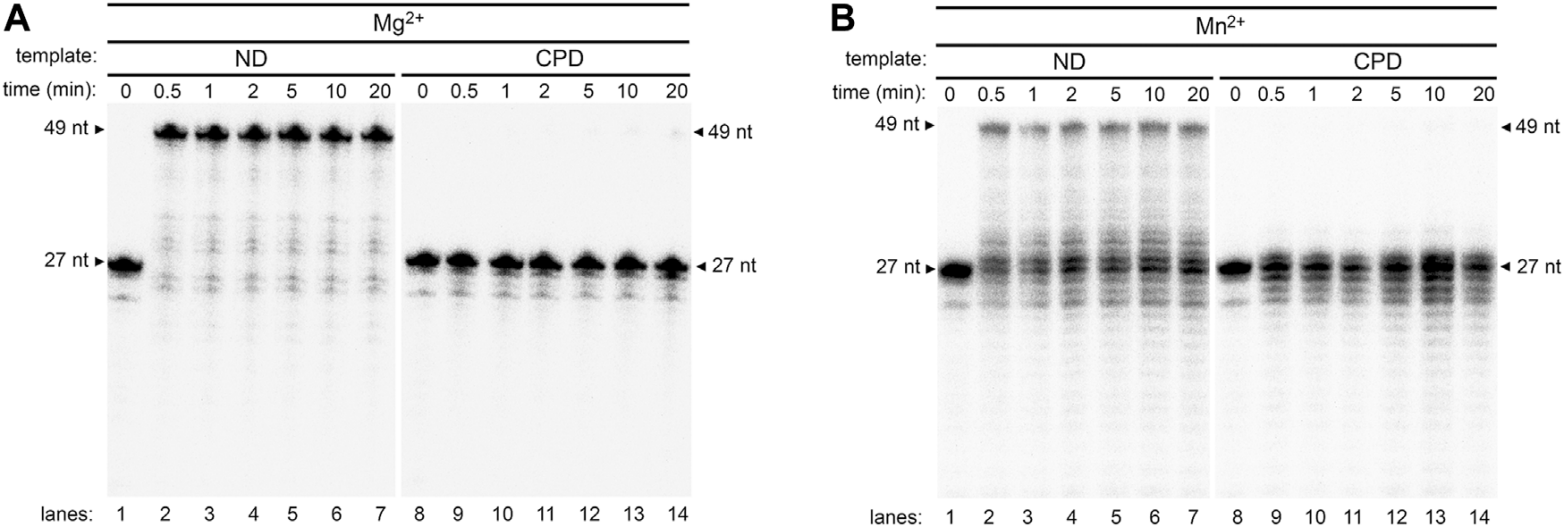
Evaluation of wild-type T7 polymerase activity on a non-damaged and CPD substrates in the presence of Mg^2+^ or Mn^2+^. **(A)** Time course activity assay of wild-type T7 DNA polymerase on T-ND (lanes 1–8) and on T-CPD (lanes 9–16) annealed to 5′- ^32^P-P_N_ in the presence of Mg^2+^. **(B)** Time course activity assay of wild-type T7 DNA polymerase on T-ND (lanes 1–8) and on T-CPD (lanes 9–16) annealed to 5′-^32^P-P_N_ in the presence of Mn^2+^.

Previous studies show that Mn^2+^ does not stimulate TLS activity in other A-family DNA polymerase members, such as *E. coli* Pol I and Klenow Fragment (Moore et al., 1981; Rabkin et al., 1983). To further evaluate the uniqueness of Mn^2+^ activity on Pol γ, we analyzed Mn^2+^ function on another A-family member, T7 DNA polymerase (T7 DNAP), a structural homolog of Pol γ. Neither Mg^2+^ nor Mn^2+^ enables wild-type T7 DNAP to bypass CPD (Figures 7A,B, *lanes* 8–14), while both metals support activity on T-ND (Figures 7A,B *lanes* 1–7). Similar to Pol γ, T7 DNAP exonuclease activity appeared to be higher in the presence of Mn^2+^ than Mg^2+^. The results suggest Mn^2+^ exclusively stimulates Pol γ′s TLS activity among replicative A-family polymerases.

## Discussion

Perhaps due to lack of UV lesion repair mechanism, mtDNA bares more pyrimidine dimers than chromosomal DNA (Birch-Machin et al., 2013). Nuclear DNA polymerases assume distinct functions in replication or lesion bypassing, but mitochondrial DNA polymerase, Pol γ, is the sole replicase responsible for synthesis on normal as well as lesion-containing mtDNA. Thus, the mitochondrial replicase will unavoidably encounter these bulky photoproducts during mtDNA replication. We present here studies of Pol γ replicating on CPD- and (6-4)PP-containing DNA templates. We showed that Pol γ exerts robust metal dependent activity on lesioned templates under physiological concentrations of Mg^2+^ and Mn^2+^ mixture or Mn^2+^ alone. The metal dependent UV dimer bypassing under a wide range of Mg^2+^ and Mn^2+^ concentrations is unique to Pol γ and has not been found in other A-family DNA polymerases.

### Pol γ May Bind to Both Mg^2+^ and Mn^2+^ in Mitochondria

Manganese is an essential element for biological functions and many ubiquitous enzymatic reactions in mitochondria. Mn^2+^ is rapidly transported into mitochondria via the uniporter with activated Ca^2+^ but slowly exported (Chance, 1965; Gavin et al., 1992), thus the organelle concentration is higher than that in cytosol. Mitochondrial concentration of Mn^2+^ is reported at 0.1–0.35 mM with an upper limit of ∼1 mM in brain, heart, and liver (Konji et al., 1985; Gunter et al., 2004), and a concentration of Mg^2+^ was 0.37–0.95 mM with upper limit of ∼1.5 mM in the heart (Corkey et al., 1986; Romani, 2011). We showed that, under the sub-stoichiometric concentrations of Mg^2+^/Mn^2+^, Pol γ displayed significant *trans*-CPD synthesis ability and presented features of binding to both metal ions. Based on these results, we hypothesize that Pol γ could bind to both metals *in vivo* and is therefore capable of replicating on non-damaged and CPD-containing templates. At very low Mg^2+^ or Mn^2+^ concentration, Pol γ is still able to replicate on both non-damaged and CPD-containing templates. Although the exact affinities of Mg^2+^ and Mn^2+^ to Pol γ are not known, it must be higher than the affinities toward dNTPs. Mg^2+^ or Mn^2+^ will first bind to metal A site in Pol γ, then Pol γ will selectively recruit incoming nucleotide pre-bound to metal ion, or metal B site. This feature of Pol γ partially compensates for lack of UV-lesion repair mechanism and TLS polymerase in mitochondria.

It is interesting to speculate Pol γ replication fidelity in the presence of Mg^2+^/Mn^2+^ mixture. At a high concentration of Mn^2+^ (above mM), many DNA polymerases become mutagenic and exhibit elevated replication errors with Mg^2+^ (Sirover and Loeb, 1976). A simplistic hypothesis would be that Mn^2+^ suppresses the proofreading exonuclease activity in DNA polymerases. However, others (Longley and Mosbaugh, 1991) and we show here that Pol γ displayed increased *exo* activity in the presence of Mn^2+^ relative to that of Mg^2+^. Thus, high concentrations in Mn^2+^-induced replication errors are not by direct inhibition of *exo* activity, rather by hindrance of error recognition. Nevertheless, at low concentrations of Mn^2+^, DNA polymerases can replicate accurately. For example, at 2 µM free Mn^2+^ concentration, *E. coli* Pol I was shown to synthesize DNA with similar error frequency as with Mg^2+^ (Beckman et al., 1985). We predict Pol γ would also replicate with high fidelity at a physiological concentration of Mn^2+^ and Mg^2+^. Needless to say, the hypothesis should be rigorously tested experimentally in future investigations. Lack of TLS activity on (6-4)PP-containing template by Pol γ suggests two possible outcomes: there may be another TLS polymerase that can overcome this UV lesion, or the damaged mtDNA is eliminated by a mitochondrial degradation mechanism as observed in *C. elegans* and primary human fibroblasts upon UV radiation damage (Bess et al., 2012; Bess et al., 2013).

### Selective Stimulation of CPD Bypassing Among Pol I Family Members

Pol γ is the only A-family polymerase found to have an intrinsic TLS activity across UV lesions as a wild-type enzyme. Studies from the Copeland and Meyer groups showed that silencing Pol γ′s exonuclease activity led to limited CPD bypassing activity, despite the diminished activity for wild-type enzymes (Miller and Grollman, 1997; Kasiviswanathan et al., 2012). We show that the TLS activities of both wild-type and exonuclease-deficient Pol γ are greatly stimulated by the presence of Mn^2+^. Additionally, plant and yeast mitochondrial DNA polymerases also exhibited TLS activity similar to human Pol γ (unpublished results). However, such activities are not found in other replicative A-family DNA polymerases. *E. coli* Pol I and Klenow Fragment lack intrinsic TLS activity and only insert one nucleotide against 3′-T in the CPD in presence of Mn^2+^ (Moore et al., 1981; Rabkin et al., 1983; Smith et al., 1998). We showed here that wild-type T7 DNAP does not display Mn^2+^-stimulated TLS activity. The Mn^2+^-stimulated TLS activity appears to be reserved only to mitochondrial DNA polymerases, perhaps to take advantage of higher Mn^2+^ concentration in the mitochondria.

Nevertheless, Mn^2+^-dependent activity is observed in certain TLS polymerases (Putrament et al., 1975; Kunkel and Loeb, 1979; Goodman et al., 1983). For example, Dpo4 bypasses abasic sites and CPD much more efficiently with Mn^2+^ than with Mg^2+^ (Vaisman et al., 2005), and DNA polymerase ι bypasses abasic sites, benzopyrenes, CPD, and (6-4)PP only in the presence of Mn^2+^ (Frank and Woodgate, 2007). In addition, PrimPol bypasses more lesions when bound to Mn^2+^ relative to Mg^2+^ (Tokarsky et al., 2017; Makarova et al., 2018).

### Manganese Ion Likely Alters Pol γ′s Pol Active Site Structure

**FIGURE 8:**
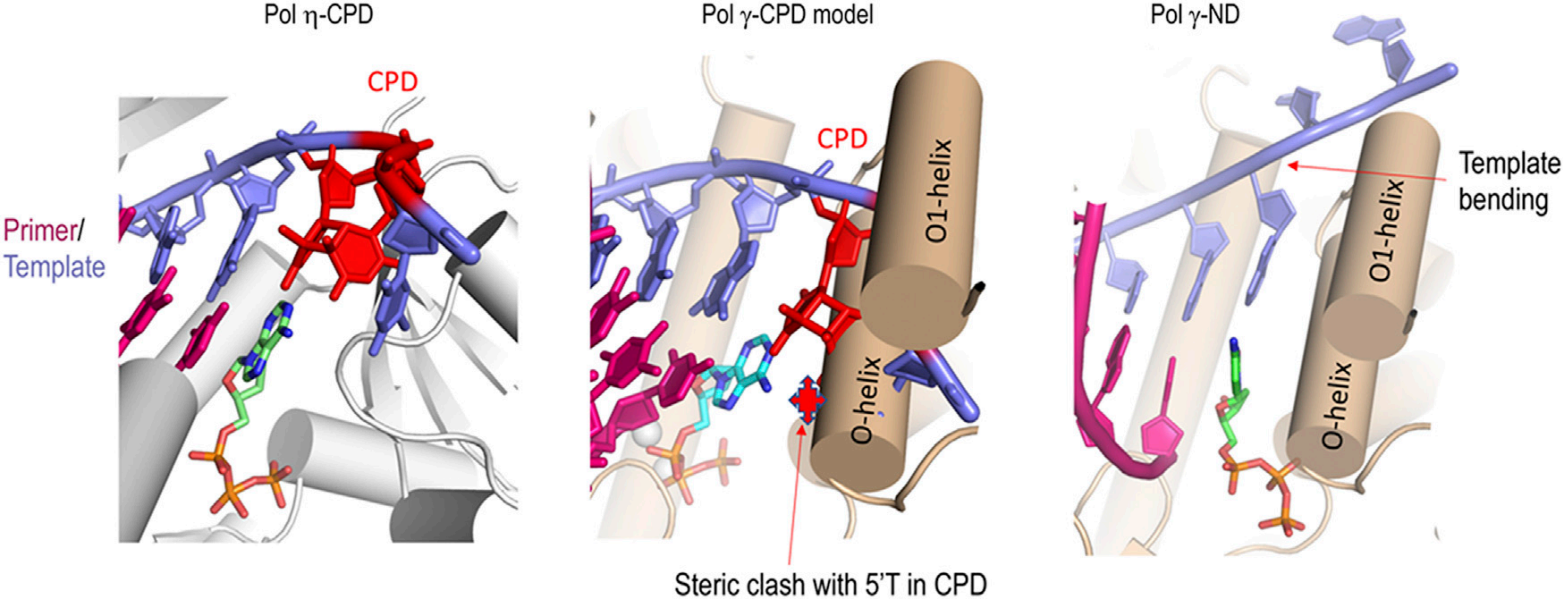
Comparison of *pol* site conformation of Pol η and Pol γ. Left, Pol *η* ternary complex with CPD (red)-containing template (blue) and primer (magenta) and incoming nucleotide (green) (PDB: 3MR3). Middle, modeled Pol γ-CPD containing primer/template DNA showing the CPD (red) steric clashes with the O helices. Right, Pol γ ternary complex with primer/template and incoming nucleotide showing the bending of the template by O- and O1-helix.

A distinct structural difference between high fidelity DNA polymerase such as Pol γ and bona fide TLS polymerases such as Pol η is the *pol* active site conformation and polymerase-enforced template DNA conformation. Crystal structures of Pol γ showed that Pol γ belongs to the A-family polymerases with characteristic fingers, thumb, and palm domains (Lee et al., 2009). Upon binding to a correct incoming nucleotide, the fingers domain undergoes large open-closed conformational changes to align the γ-phosphate of dNTP, 3′-OH of the primer, and catalytic metal ions for optimal catalysis for phosphodiester bond formation (Lee et al., 2009; Szymanski et al., 2015; Sohl et al., 2015). Significantly, the template bends 90° at the coding base (*n*) and downstream neighboring residue (*n+1*) (Figure 8, right). DNA template bending in Pol γ is accomplished by the helices O and O1 in the finger domain. Such template strand bending is thought to prevent template ‘slippage’ and ensuring replication fidelity. A non-bendable dimer is deterred from entering the *pol* site and stalls replication (Li et al., 2004). Indeed, the structure of T7 DNA polymerase, a Pol γ homolog, complexed with CPD lesion (Li et al., 2004) shows that after incorporating a nucleotide opposing the 3′-T of CPD, base pairing with the 5′-T of CPD is severely crooked, preventing replication at the *n+1* position.

Contrary to the closed *pol* site in high-fidelity polymerases ternary conformation, the *pol* active site is more open in Y-family TLS polymerases Pol *η*. Structure of DNA polymerase *η* (Pol η) complexed with a CPD-containing template and primer duplex reveals that the bulky CPD can be easily accommodated in the *pol* site due to the preformed, spacious *pol* active site and highly mobile little-fingers and thumb domains, shared features in Y-family DNA polymerases (Figure 8, left) (Yang and Woodgate, 2007; Biertümpfel et al., 2010). Unlike replicative DNA polymerases, the fingers subdomain in Y-family DNA polymerases does not go through large conformational changes. Rather, the enzyme is already poised for DNA synthesis even in the absence of incoming nucleotides. Little-finger and thumb domains go through the largest conformational changes and seem to confer specific TLS activities (Boudsocq et al., 2004; Yang and Woodgate, 2007).

**FIGURE 9:**
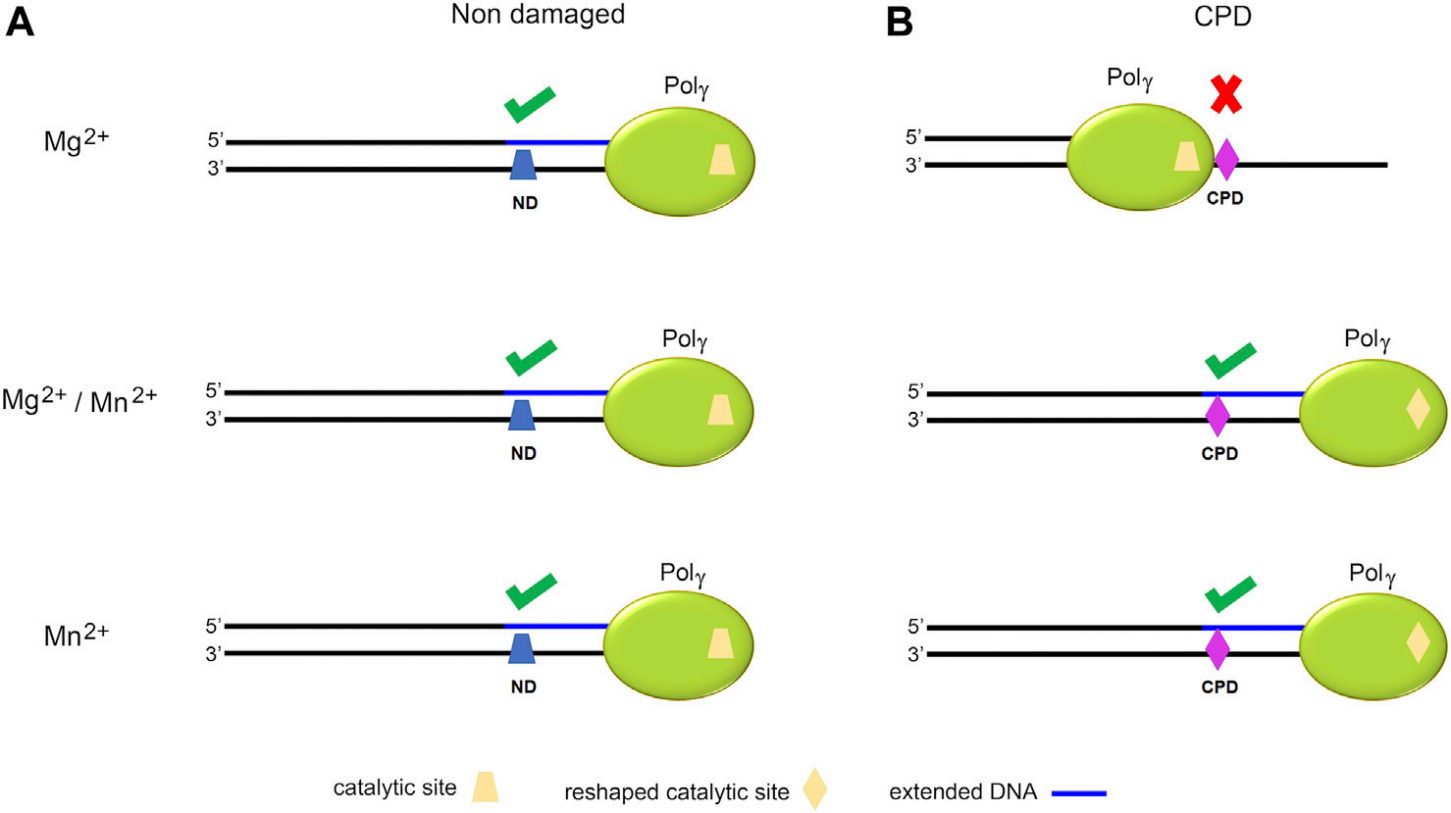
Schematic model of the hypothetical function of Mg^2+^ and Mn^2+^ in reshaping Pol γ′s active site. Illustration of Pol γ (green) on an ND- (blue trapezoid) or CPD-containing template (violet rhomboid). **(A)** Representation of DNA replication by Pol γ on an ND-containing substrate in the presence of Mg^2+^, Mg^2+^/Mn^2+^, or Mn^2+^ showing full extension in all cases without significant changes in the catalytic site (yellow trapezoid). **(B)** Representation of DNA replication by Pol γ on a CPD-containing template illustrating a change of the Pol γ′s catalytic site (yellow trapezoid) in the presence of Mn^2+^ upon encountering CPD to trigger TLS activity.

To provide a structural interpretation for Pol γ metal-dependent TLS activity, we composited a ternary complex of Pol γ, primer/CPD-template, and an incoming nucleotide by docking CPD-containing substrate from T7 DNA polymerase complex (PDB: 1SL2) onto Pol γ ternary complex (PDB: 4ZTZ) after superposition on the two *pol* active sites (Figure 8, middle)*.* The resulting complex showed that the CPD would sterically clash with the O1 helix fingers domain severely if no conformational change occurs. For Pol γ to accommodate the bulky CPD, the fingers domain would have to adopt a more open configuration resembling the Pol γ-DNA binary complex. In addition, Mn^2+^ may also enlarge the *pol* site in a way that no longer bends the template strand, allowing the bulky thymine dimers to enter effortlessly. We thus hypothesize that Mn^2+^ may alter Pol γ structure from high-fidelity configuration to that of TLS polymerases. Under this idea, when Pol γ replicates on a non-damaged DNA, the catalytic site is not challenged for which a catalytic site reshaping is not needed (Figure 9A). But on a CPD-containing substrate, due to its volume, Pol γ′s catalytic site is reshaped in the presence of Mn^2+^ or a mixture with Mg^2+^ (Figure 9B). More studies should be addressed to better understand this mechanism.

Pol ι, another Y-family DNA polymerase, has better TLS activity across UV lesions in the presence of Mn^2+^ compared to Mg^2+^ (Frank and Woodgate, 2007). The mechanism behind Mn^2+^-mediated TLS in Pol ι is not by large conformational changes of protein. Crystal structures of Pol ι do not display noticeable overall structural changes in the presence of Mn^2+^ compared to Mg^2+^ except for small changes near divalent cations in the active site (Choi et al., 2016). Mn^2+^ supports more optimal octahedral coordination compared to Mg^2+^, explaining the higher activity and fidelity in presence of Mn^2+^. In addition, Pol ι does not go through large conformational change upon dNTP binding like replicative DNA polymerases do, which gives molecular explanation behind its ability to tolerate bulkier lesions in its wide and rigid active site (Nair et al., 2006). For replicative polymerases like Pol γ, finger domain movement upon dNTP binding is essential. It is possible that Mn^2+^ binding induces sufficient structural changes to accommodate CPD.

Mn^2+^-induced conformational changes are seen in other polymerases. The *pol* active site of Pol *η* widens when bound to Mn^2+^ relative to being bound to Mg^2+^ and Ca^2+^ (Yang and Woodgate, 2007; Ling et al., 2003; Rechkoblit et al., 2006; Weng et al., 2018). The proteolysis pattern for a catalytic subunit of herpes simplex virus DNA polymerase (UL30) bound to platinated DNA differs in the presence of Mn^2+^ than in the presence of Mg^2+^, implying that UL30 undergoes conformational changes between Mn^2+^ to Mg^2+^ bound states (Villani et al., 2002). If Mn^2+^ actually alters Pol γ *pol* site and the fingers domain resemble that in Pol η, Pol γ would be able to accommodate the rigid thymine dimer in the enlarged *pol* site and to incorporate incoming nucleotide opposite to the dimer without stringent geometry check for W-C base pairing complementarity. Crystal structures of RB69 DNA polymerase, replicative B-family DNA polymerase, in the presence of Mg^2+^ or Mn^2+^ do not display significant conformational changes (Xia et al., 2011). It is likely the same for Pol γ when it is replicating on T-ND. However, when Pol γ is challenged with bulky lesion like CPD, the Mn^2+^-mediated flexibility in the *pol* site may allow enlargement to accommodate CPD (Figure 8). Currently, structural studies revealed only two catalytic metal ions in the *pol* site; however, EPR study of *E. coli* Pol I showed at least 25 Mn^2+^ binding sites were present (Slater et al., 1972). Whether the catalytic metal ions or additional metal ions are involved in reshaping the *pol* site warrants further investigation.

## Methods

### Proteins Preparation

His-tagged Pol γA and Pol γB subunits were expressed in Sf9 insect cells and *E. coli* Rosetta (DE3) cells, respectively, and purified as previously described (Lee et al., 2009). Wild-type Pol γA subunit and exonuclease-deficient (*exo*-) variant containing double mutation D198A/E200A lack N-terminal 25 residues (the putative mitochondrial localization sequence) as well as 10 of the 13 sequential glutamines (residues 43–52). Briefly, both Pol γA wild-type and exonuclease deficient variant were purified sequentially using TALON (Cytiva, Marlborough, MA) and gel filtration column Superdex 200. Pol γB subunit lacking N-terminal 25 residues was purified using Ni-NTA (Qiagen, Germantown, MD) and Mono S affinity chromatography. Purified Pol γA and Pol γB were complexed at 1:2 M ratio, respectively, then applied to a gel filtration column Superdex 200. After gel filtration, pure fractions were pooled and concentrated in a buffer containing 20 mM HEPES (pH 7.5), 140 mM KCl, 5% glycerol, 1 mM EDTA (pH 8,0), and 10 mM BME. Fractions were aliquoted and stored at −80°C.

Wild-type T7 DNA polymerase bound to thioredoxin was purchased from New England Biolab (Ipswich, MA). Concentrations of the proteins were determined on the NanoDrop™ spectrophotometer (Thermo Scientific, Waltham, MA) based on the absorbance at 280 nm. All experiments reported in this study were carried out with the same batch purified proteins. The protein concentrations are determined by A280 absorption and specific extinction coefficients, not active site concentration. The active site concentrations of purified Pol γ are routinely measured to be 80–84%.

### DNA Substrates

All oligonucleotides are synthesized by Integrated DNA Technologies (Morrisville, NC) except for templates containing CPD and (6-4)PP (Table 1). The oligonucleotides containing the CPD and the (6-4)PP were synthesized as described previously (Murata et al., 1990; Iwai et al., 1996). Complementary oligonucleotides were mixed in solution containing 50 mM Tris (pH 7.8), 50 mM NaCl, and 1 mM EDTA (pH 8), then were annealed by heating at 95°C for 5 min followed by slowly cooling to room temperature overnight. For all activity assays, 5′-end of the primer strands were labeled using T4 polynucleotide kinase (New England Biolab, Ipswich, MA) using γ-^32^P-ATP (PerkinElmer, Waltham, MA).

### Polymerase Activity Assays

Three primer/template duplexes were formed by primer P_N_ (27 nt) annealed to a 49 nt non-damaged template (T-ND), a CPD-containing template (T-CPD), or a (6-4)PP-containing template (T-(6–4)PP) (Table 1). For UV product-containing DNA, 200 nM wild-type or exonuclease-deficient Pol γ was pre-incubated with 1000 nM P_N_/T-CPD or P_N_/T-(6–4)PP in Buffer N (25 mM HEPES pH 7.5, 140 mM KCl, 1 mM EDTA, 5% glycerol, and 100 μg/ml BSA) at 37°C for 5 min. For T-ND, 100 nM wild-type or exonuclease-deficient Polγ was preincubated with 1000 nM P_N_/T-ND in Buffer N. The reaction was started by the addition of equal volume of Buffer C (Buffer N supplemented with 400 µM dNTP mix and 40 mM MgCl_2_ or MnCl_2_) to a final concentration of 200 µM dNTP mix and 20 mM MgCl_2_ or MnCl_2_. Reaction was quenched at the indicated times by adding ninefold excess of Buffer Q (80% Formamide, 50 mM EDTA pH 8, 0.1% SDS, 5% glycerol, and 0.02% bromophenol blue). Quenched samples were heated at 95°C for 5 min and resolved on a 23% polyacrylamide gel containing 7 M urea. Gels were soaked in solution containing 50% methanol and 20% glycerol prior to drying overnight under vacuum at 50°C. Gels were exposed on a phosphor screen, which was imaged using Amersham Typhoon RGB scanner (Cytiva, Marlborough, MA). The intensities of bands were quantified using ImageQuant TL 8.2 (Cytiva, Marlborough, MA). Graphs were plotted using the mean and standard deviation in SigmaPlot 14 (Systat Software, San Jose, CA). To obtain the rate of full-length product formation, we used the following equation: $rate=\frac{I_{FL}}{I_{0}}$ , where *I*_*FL*_ is the intensity of the full-length product band at 120 s, and *I*_*0*_ is the intensity of the original substrate band at 0 s. We normalized the enzyme concentration as polymerase activity assays on T-CPD contained 100 nM Pol γ whereas polymerase activity assays on T-ND contained 50 nM Pol γ.

For polymerase activity assays using wild-type T7 DNA polymerase, a total of 400 nM T7 DNA polymerase was pre-incubated with 200 nM of P_N_/T-ND or P_N_/T-CPD in Buffer N at 37°C for 5 min. The reaction was started by addition of equal volume of Buffer C to a final concentration of 200 µM dNTP mix and 20 mM MgCl_2_ or MnCl_2_. Quench step, electrophoresis, and autoradiography were carried out identically to polymerase activity assays using P_N_.

### Exonuclease Assays

A total of 400 nM Polγ was pre-incubated with 1000 nM single stranded primer P_N_ or duplex P_N_/T-ND in Buffer N, and the reaction was started by addition of an equal volume of Buffer C without dNTPs to a final concentration of 20 mM MgCl_2_ or MnCl_2_. Quench step, electrophoresis, autoradiography, and quantification were carried out identically to polymerase activity assays. The experimental data were fitted to an equation $y=Ae^{-kt}$ , where *A* is the amplitude and *k* is the rate of excision. Graphs were plotted using the mean and standard deviation in SigmaPlot 14 (Systat Software, San Jose, CA).

### Polymerase Assays With Mixed Metal Ions

For polymerase assay using mixed metal ions, 1000 nM wild-type Polγ was pre-incubated with 1000 nM P_N_/T-ND or P_N_/T-CPD in Buffer N at 37°C for 5 min. The reaction was started by addition of an equal volume of Buffer C to final concentrations of either constant 1 mM MgCl_2_ and varied MnCl_2_ (0.1, 0.2, 0.3 0.4, 0.5, 1, and 10 mM) concentrations, or constant 0.4 mM MnCl_2_ and varied MgCl_2_ (0.1, 0.2, 0.4, 0.4, 0.5, 1, and 10 mM) concentrations. Reaction was quenched 5 min after adding ninefold excess of Buffer Q. Electrophoresis, autoradiography, and quantification were carried out identically to polymerase activity assays.

### Polymerase Assays With Single Metal Ions

For polymerase assay using single metal ions, 200 nM wild-type Polγ was pre-incubated with 200 nM P_N_/T-ND or P_N_/T-CPD in Buffer N at 37°C for 5 min. Reaction was started by addition of an equal volume of Buffer C containing MgCl_2_ or MnCl_2_ (0.025, 0.05, 0.1 0.2, 0.4, 0.8, 1.0, and 10 mM). Reaction was quenched 5 min after adding ninefold excess of Buffer Q. Electrophoresis, autoradiography, and quantification were carried out identically to polymerase activity assays.

### Experimental Replicas

Experiments with corresponding graphs containing error bars were repeated three times. The rest of the experiments with corresponding graphs not containing error bars were repeated twice.

## Data Availability

The data supporting the conclusions of this article will be made available by the corresponding authors, without undue reservation.
